# Glutathione Depletion Exacerbates Hepatic *Mycobacterium tuberculosis* Infection

**DOI:** 10.3390/biology14020131

**Published:** 2025-01-27

**Authors:** Kayvan Sasaninia, Aishvaryaa Shree Mohan, Ali Badaoui, Ira Glassman, Sonyeol Yoon, Arshavir Karapetyan, Afsal Kolloli, Ranjeet Kumar, Santhamani Ramasamy, Selvakumar Subbian, Vishwanath Venketaraman

**Affiliations:** 1 College of Osteopathic Medicine of the Pacific, Western University of Health Sciences, Pomona, CA 91766, USA; kayvan.sasaninia@westernu.edu (K.S.); aishu.mohan5@gmail.com (A.S.M.); ali.badaoui@westernu.edu (A.B.); ira.glassman@westernu.edu (I.G.); sonyeol.yoon@westernu.edu (S.Y.); arshavir.karapetyan@westernu.edu (A.K.); 2 Public Health Research Institute—New Jersey Medical School, Rutgers University, Newark, NJ 07103, USA; ak1482@njms.rutgers.edu (A.K.); rk879@njms.rutgers.edu (R.K.); santhuvet@gmail.com (S.R.); subbiase@njms.rutgers.edu (S.S.)

**Keywords:** *Mycobacterium tuberculosis*, glutathione, oxidative stress, granuloma, extrapulmonary tuberculosis, inflammation

## Abstract

Extrapulmonary tuberculosis (EPTB) accounts for approximately 17% of all *Mycobacterium tuberculosis* (*M.tb*) infections globally. Immunocompromised individuals, such as those with HIV infection or type 2 diabetes mellitus (T2DM), are at an increased risk for EPTB. Previous studies have demonstrated that patients with HIV and T2DM exhibit diminished synthesis of glutathione (GSH) synthesizing enzymes. In a murine model, we showed that the diethyl maleate (DEM)-induced depletion of GSH in the lungs led to increased *M.tb* burden and an impaired pulmonary granulomatous response to *M.tb* infection. However, the effects of GSH depletion during active EPTB in the liver and spleen have yet to be elucidated. In this study, we evaluated hepatic GSH and malondialdehyde (MDA) levels, as well as cytokine profiles, in untreated and DEM-treated *M.tb*-infected wild-type (WT) C57BL/6 mice. Additionally, we assessed hepatic and splenic *M.tb* burdens and tissue pathologies. DEM treatment resulted in a significant decrease in the levels of the reduced form of GSH and an increase in MDA, oxidized GSH, and interleukin (IL)-6 levels. Furthermore, DEM-induced GSH decrease was associated with decreased production of IL-12 and IL-17 and elevated production of interferon-gamma (IFN-γ), tumor necrosis factor (TNF)-α, and transforming growth factor (TGF)-β. A significant increase in *M.tb* growth was detected in the liver and spleen in DEM-treated *M.tb*-infected mice. Large, disorganized lymphocyte infiltrates were detected in the hepatic tissues of DEM-treated mice. Overall, GSH diminishment impaired the granulomatous response to *M.tb* in the liver and exacerbated *M.tb* growth in both the liver and spleen. These findings provide critical insights into the immunomodulatory role of GSH in TB pathogenesis and suggest potential therapeutic avenues for the treatment of extrapulmonary *M.tb* infections.

## Simple Summary

Extrapulmonary tuberculosis (EPTB) makes up 17% of all tuberculosis cases worldwide. Immunocompromised patients, such as those with HIV or type 2 diabetes, are more likely to obtain EPTB. Studies show that these individuals often have lower levels of glutathione (GSH), a key antioxidant. In experiments with mice, depleting GSH in the lungs led to more severe tuberculosis infections. This study examined how GSH depletion affects tuberculosis in the liver and spleen. Mice with diminished GSH levels had higher levels of oxidative stress, inflammation, and *M.tb* burden in the liver and spleen. These findings highlight the important role of GSH in modulating the granulomatous response against EPTB.

## 1. Introduction

*Mycobacterium tuberculosis* (*M.tb*) is known to cause tuberculosis (TB), a primary pulmonary disease, and extrapulmonary diseases in up to 17% of all cases [1]. Disseminated TB refers to the involvement of at least two non-contiguous body organs, blood, bone marrow, or liver. Individuals at the highest risk of TB infection include those from endemic countries, those with excessive alcohol and/or tobacco use, and immunocompromised individuals like those with human immunodeficiency virus (HIV)/acquired immunodeficiency syndrome (AIDS), immunosuppressant usage, and diabetes [2]. These individuals are especially susceptible to infection due to the dysregulation of T-helper cells, which normally upregulate macrophages to contain *M.tb* in granulomas [3]. If cell-mediated immunity fails, *M.tb* can seed through the lymphatic system and reach the arterial system to spread to other organs, like the liver. Risk factors for extrapulmonary dissemination and mortality of tuberculosis include age above 45 years, female gender, HIV status, end-stage renal disease, and excessive alcohol use within the past 12 months [4].

For TB to disseminate to organs outside the pulmonary system, *M.tb* must traverse to the lymphatic system and/or bloodstream. Pathogenic mycobacterium can travel across alveolar epithelial cells inside phagocytic cells such as macrophages or spread as free bacteria [5]. Once inside lymph nodes or the bloodstream, *M.tb* may seed secondary lymphoid organs such as the spleen or liver [5]. Both the innate and adaptive immune systems play a role in controlling TB to prevent dissemination. Impaired T-cell immunity alters granuloma formation and reduces IFN-gamma (IFN-γ) expression [6].

Immunocompromised patients represent a highly susceptible population disproportionately affected by both common and opportunistic pathogens. Studies have demonstrated that immunocompromised patients, such as those with HIV or type 2 diabetes, have low levels of glutathione (GSH) and high levels of oxidative stress [7,8]. GSH is a ubiquitous antioxidant essential for maintaining cellular redox homeostasis, providing host cell protection from reactive oxygen species (ROS) or reactive nitrogen species (RNS) [9]. Emerging evidence suggests oxidative stress and GSH deficiency facilitate the pulmonary spread of *M.tb* [10,11]. *M.tb*-induced macrophage necrosis and ferroptosis were associated with reduced levels of GSH and glutathione peroxidase expression [10]. Deficiency in glutathione and glutathione peroxidase in mice has demonstrated increased susceptibility to *M.tb* infection [11]. We have previously demonstrated that GSH depletion is associated with a significant increase in *M.tb* bacterial load and survival in the lungs of *M.tb*-infected mice [12]. We have also demonstrated that GSH depletion interferes with granuloma formation in the lungs of infected mice, leading to poorly defined pulmonary granulomas [12]. However, we have yet to elucidate the effects of GSH depletion in extrapulmonary sites such as the liver and spleen and its effect on the granulomatous response against *M.tb*.

This study aims to evaluate the effects of GSH depletion in the livers of *M.tb*-infected mice. We utilized diethyl maleate (DEM), a known GSH depletion agent, to induce GSH deficiency in mice. In this study, we evaluated hepatic GSH and malondialdehyde (MDA) levels, as well as cytokine profiles, in untreated and DEM-treated *M.tb*-infected wild-type (WT) C57BL/6 mice. Additionally, we assessed hepatic and splenic *M.tb* burdens and tissue pathologies. The findings of this study offer insight into the role of GSH in the modulation of the host immune response against *M.tb* infection.

## 2. Materials and Methods

### 2.1. Bacteria and Chemicals

*Mycobacterium tuberculosis* H37Rv strain was used for mice infection. In brief, the bacteria were cultivated until reaching OD600 = 0.6 to 0.8 in Middlebrook 7H9 medium (Difco BD, Franklin Lakes, NJ, USA) supplemented with 10% albumin dextrose complex (ADC) enrichment (Difco BD, Franklin Lakes, NJ, USA). H37Rv culture was grown to the logarithmic phase, achieving an OD600 value between 0.6 and 0.8. The culture was then harvested by centrifugation at 3000× *g* rpm. The bacterial pellet was vortexed with 3 mm sterile glass beads to disperse clumps, and larger clumps were removed using a 5 µm syringe filter. Bacterial concentration was determined using a CFU assay. Serial dilutions of the culture were plated on Middlebrook 7H10 agar and incubated at 37 °C with 5% CO_2_ for 3–4 weeks. The CFU count was recorded. *M.tb* stocks were then divided into aliquots and stored at −80 °C. Thawed stock vials were diluted to prepare the infection inoculum, following previously outlined methods [12,13]. Chemicals were sourced from Millipore Sigma (Millipore Sigma, Burlington, MA, USA) unless otherwise stated.

### 2.2. Aerosol Infection of Mice, Treatment, and Bacterial CFU Assay

All animals in the study were humanely handled in accordance with National Institute of Health (NIH) guidance procedures. The animal protocols were performed in biosafety level 3 facilities as per the approved procedures of the Rutgers University Institutional Animal Care and Use Committee (IACUC). This study was approved by the IACUC (Protocol#R19 IACUC008).

Wild-type (WT) C57Bl/6 mice aged 6–8 weeks were procured from Jackson Laboratories (Bar Harbor, ME, USA). The *M.tb* inoculum for infecting mice was prepared according to established protocols [13]. The mice were exposed to *M.tb* aerosols using a Madison Chamber (Glas-Col LLC, Madison, WI, USA) optimized to deliver a standard low dose of approximately 100 CFU, as previously reported [12,13]. Liver samples were collected, and approximately 40% of each liver was homogenized in 2 mL of sterile 1xPBS, serially diluted, and plated on Middlebrook 7H11 agar media (Difco BD, Franklin Lakes, NJ, USA). The number of bacterial colonies was counted after incubating the plates for 4–6 weeks at 37 °C with 5% CO_2_ supply.

Following infection, mice were randomly assigned to two groups: (1) no treatment or (2) treatment with DEM (50 mM in water). All treatments commenced on the day of infection and continued until the experimental endpoint (8 weeks post-infection). DEM was administered via daily oral gavage. At specific time intervals (2, 4, and 8 weeks post-infection), three mice from each group were euthanized. Standard necropsies were performed, and liver samples were harvested as previously described [12]. Samples were homogenized and plated to enumerate bacterial load post-euthanasia.

For histological analysis, the lower lobe of the liver was fixed in 10% neutral buffered formalin. The remaining liver lysates were filtered through a 0.2-micron filter and utilized for downstream cytokine, MDA, and GSH analyses.

### 2.3. Histology Staining of Liver Sections and Morphometry

Sections of the liver fixed in 10% neutral formalin solution were embedded in paraffin, sliced into 5 µm sections, and stained with hematoxylin and eosin (H&E) to visualize granuloma organization and leukocyte distribution. Analysis of the stained sections was conducted using an Olympus Model BX41TF microscope, and images were captured using an Olympus DP Controller (Tokyo, Japan).

### 2.4. Quantification of Glutathione Levels

GSH levels were assessed in liver homogenates from untreated and DEM-treated mice at 2 weeks, 4 weeks, and 8 weeks post-infection, using a Glutathione Colorimetric Detection Kit from Invitrogen (Cat. # EIAGSHC) and following the manufacturer’s protocol (Thermo Fisher Scientific, Waltham, MA, USA). The reduced GSH (rGSH) was calculated by subtracting oxidized GSH (GSSG) from the total GSH. All measurements were standardized to the total protein levels in the samples, and the results were expressed as micromolar GSH per microgram of protein.

### 2.5. Cytokine Measurement

IFN-γ, IL-2, TNF-α, IL-6, IL-12, IL-17, and TGF-β levels were assessed in liver homogenates from untreated and DEM-treated mice using enzyme-linked immunosorbent assay (ELISA) kits obtained from Thermofisher Scientific (Thermofisher Scientific, Waltham, MA, USA). Specifically, the following ELISA kits were used: IL-12 p70 Mouse Uncoated ELISA Kit (Cat # 88–7,121–88), IFN-γ Mouse Uncoated ELISA Kit (Cat. # 88–7,314–88), IL-2 Mouse Uncoated ELISA Kit (Cat # 88–7,024–88), TNF-α Mouse Uncoated ELISA Kit (Cat. # 88–7,324–88), IL-17A (homodimer) Mouse Uncoated ELISA Kit (Cat # 88–7,371–86), IL-6 Mouse Uncoated ELISA Kit (Cat # 88–7,064–88), and Human/Mouse TGF-β1 Uncoated ELISA Kit (Cat. # 88–8,350–88). Cytokine levels were determined following the manufacturer’s instructions. All measurements were normalized to the total protein levels in the samples, and the results were reported as picograms of cytokine per microgram of protein.

### 2.6. Statistical Analysis

Statistical analysis was conducted using GraphPad Prism Software 8. For comparisons involving two groups, an unpaired *t*-test with Welch’s correction was applied. For comparisons involving multiple groups, the Kruskal–Wallis test was applied. Reported values represent means and standard deviations for each category, with *p*-values < 0.05 considered statistically significant. A dot indicates a replicate. An asterisk (*) indicates a direct comparison to the previous category with a *p*-value below 0.05. Two asterisks (**) imply a *p*-value below 0.005, three asterisks (***) suggest a *p*-value < 0.0005, and four asterisks (****) indicate a *p*-value < 0.0001.

## 3. Results

### 3.1. rGSH Levels Are Decreased, and GSSG Levels Are Elevated in the Liver Post-DEM Treatment

**Figure 1:**
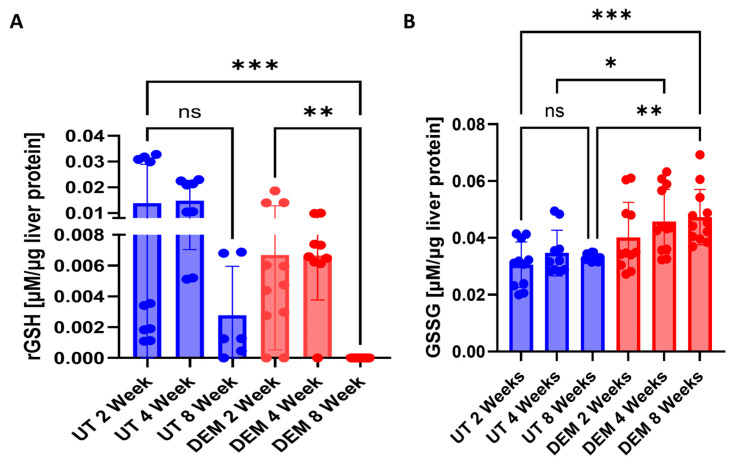
Levels of a reduced form (rGSH) and oxidized form (GSSG) of glutathione in the liver of untreated (n = 3) and DEM-treated (n = 3) *M.tb*-infected C57BL/6 mice. (**A**) rGSH levels 2, 4, and 8 weeks post-*M.tb* infection; (**B**) GSSG levels 2, 4, and 8 weeks post-*M.tb* infection. The sample size (n) includes three female mice (n = 3) each in the untreated and DEM-treated groups. Dots indicate replicates of each mouse. Comparisons between untreated and DEM-treated groups were analyzed via GraphPad prism software using the Kruskal–Wallis test. The placement of an asterisk (*) indicates statistical significance between compared groups. A single asterisk (*) denotes a *p*-value < 0.05. Double asterisks (**) imply a *p*-value below 0.005. Triple asterisks (***) indicate a *p*-value < 0.0005. ns indicates no significance.

A two-fold decrease in the reduced form of GSH (rGSH) levels was detected 2 weeks post-*M.tb* infection after DEM treatment (Figure 1A). DEM treatment also led to a 3-fold decrease in rGSH levels at 4 weeks post-*M.tb* infection (Figure 1B). After 8 weeks of *M.tb* infection, rGSH levels were almost undetectable in the DEM treatment group (Figure 1A). DEM treatment of *M.tb*-infected mice caused a significant increase in the oxidized form of GSH (GSSG) levels at 4 weeks and 8 weeks post-infection and treatment compared to untreated mice (Figure 1B). Compared to the untreated group, there was a 30% increase in the levels of the GSSG in the DEM-treated group at all the tested time points. When evaluating rGSH levels over time, untreated mice demonstrated a nonsignificant reduction in rGSH at 8 weeks post-*M.tb* infection compared to untreated mice infected at 2 weeks *M.tb* post-infection. However, DEM-treated mice demonstrated a significant reduction at 8 weeks post-infection in rGSH levels compared to DEM-treated mice 2 weeks post-infection and untreated mice 2 weeks post-infection. GSSG levels over time showed no significant changes in untreated mice 8 weeks post-infection. A nonsignificant increase in GSSG was observed 8 weeks post-infection compared to 2 weeks post-infection in DEM-treated mice. A significant increase was observed in DEM-treated mice 8 weeks post-infection compared to untreated mice 2 weeks post-*M.tb* infection.

### 3.2. MDA Levels Are Increased in the Liver Post-DEM Treatment

**Figure 2:**
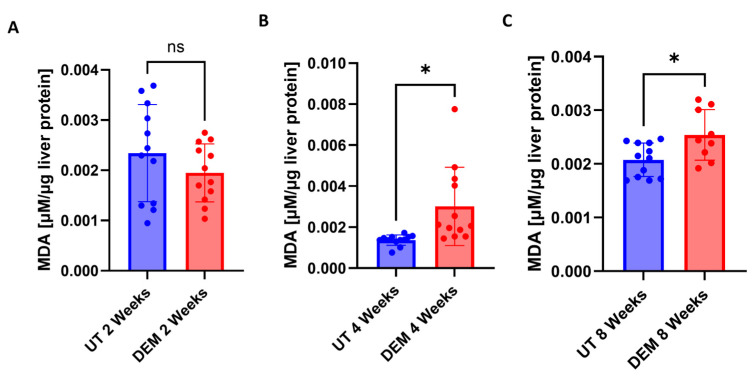
Malondialdehyde (MDA) levels in the liver of untreated and DEM-treated C57BL/6 mice; (**A**) 2 weeks post-*M.tb* infection, (**B**) 4 weeks post-*M.tb* infection, and (**C**) 8 weeks post-*M.tb* infection. The sample size (n) includes three female mice (n = 3) each in the untreated and DEM-treated groups. Dots indicate replicates of each mouse. Comparisons between untreated and DEM-treated groups were analyzed via GraphPad prism software using an unpaired *t*-test with Welch’s correction. The placement of an asterisk (*) indicates statistical significance between compared groups with *p*-value < 0.05. ns indicate no significance.

Malondialdehyde is a stable end product of lipid peroxidation and is used to measure oxidative stress. No significant changes were detected 2 weeks post-infection in DEM treatment (Figure 2A). DEM treatment of *M.tb*-infected mice caused a significant increase in the levels of MDA at 4 weeks post-infection and treatment (Figure 2B). MDA levels were significantly elevated 8 weeks post-infection in DEM-treated mice compared to untreated mice (Figure 2C).

### 3.3. DEM Treatment Altered Cytokine Production in the Liver

**Figure 3:**
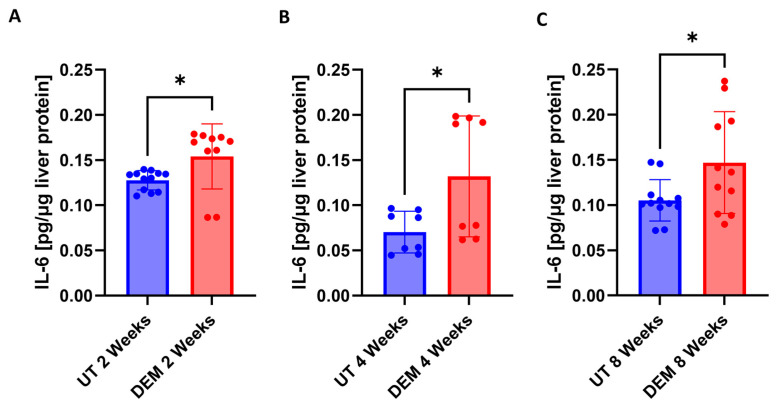
Levels of IL-6 in the liver of untreated and DEM-treated C57BL/6 mice; (**A**) 2 weeks post-*M.tb* infection, (**B**) 4 weeks post-*M.tb* infection, and (**C**) 8 weeks post-*M.tb* infection. The sample size (n) includes three female mice (n = 3) each in the untreated and DEM-treated groups. Dots indicate replicates of each mouse. Comparisons between untreated and DEM-treated groups were analyzed via GraphPad prism software using an unpaired *t*-test with Welch’s correction. The placement of an asterisk (*) indicates statistical significance between compared groups. A single asterisk (*) denotes a *p*-value < 0.05.

To assess the host immune effects against oxidative stress following DEM treatment, IL-6 levels were assessed. A significant increase in IL-6 levels was detected in *M.tb*-infected, DEM-treated mice at 2 weeks post-infection (Figure 3A). After 4 weeks of *M.tb* infection, DEM treatment significantly increased IL-6 levels (Figure 3B). Similarly, IL-6 levels remained significantly elevated at 8 weeks post-*M.tb* infection and DEM treatment (Figure 3C).

**Figure 4:**
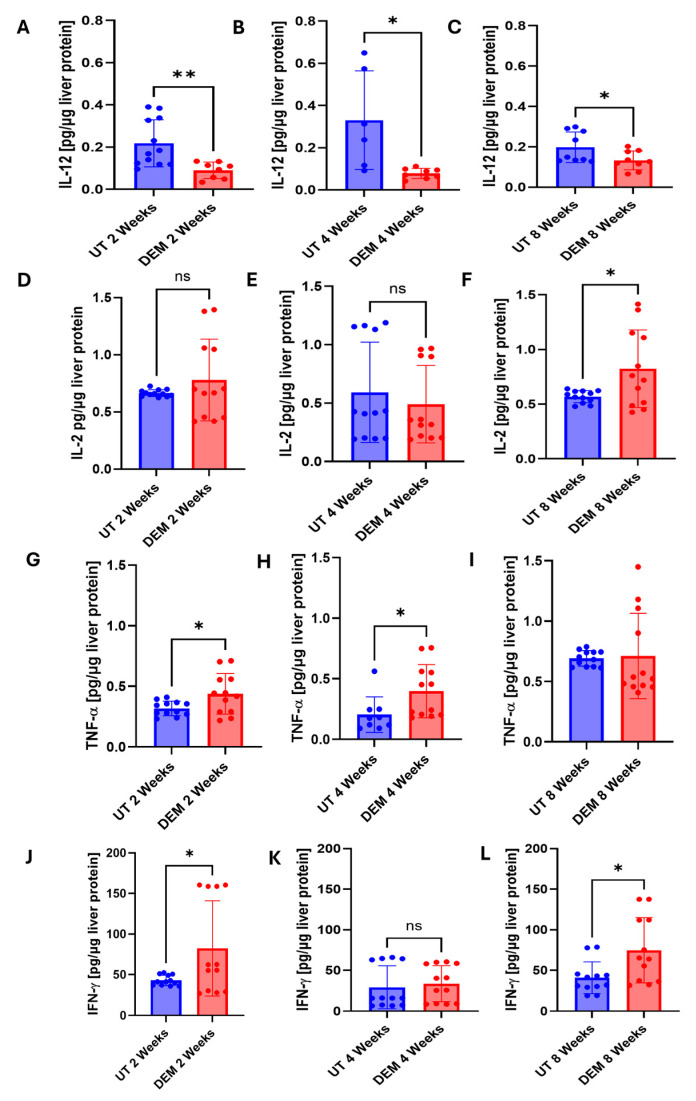
Levels of Th1 cytokines in the liver of untreated and DEM-treated *M.tb*-infected C57BL/6 mice. (**A**–**C**) IL-12 levels 2, 4, and 8 weeks post-*M.tb* infection; (**D**–**F**) IL-2 levels 2, 4, and 8 weeks post-*M.tb* infection; (**G**–**I**) TNF-α levels 2, 4, and 8 weeks post-M.tb infection; (**J**–**L**) IFN-γ levels 2, 4, and 8 weeks post-*M.tb* infection. The sample size (n) includes three female mice (n = 3) each in the untreated and DEM-treated groups. Dots represent replicates of each mouse. Comparisons between untreated and DEM-treated groups were analyzed via GraphPad prism software using an unpaired *t*-test with Welch’s correction. The placement of an asterisk (*) indicates statistical significance between compared groups. A single asterisk (*) denotes a *p*-value < 0.05. Double asterisk (**) indicates a *p*-value < 0.005. ns indicates statistical non-significance.

**Figure 5:**
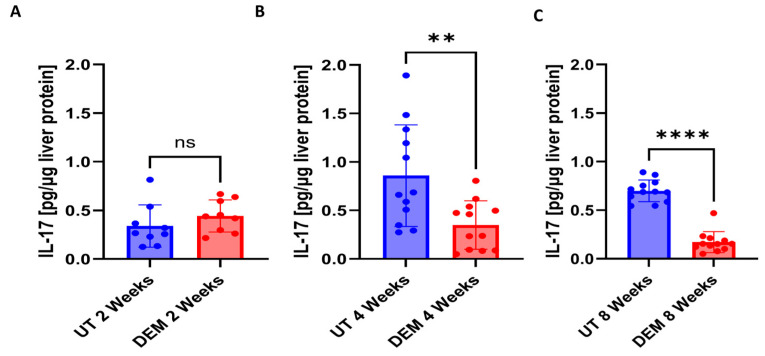
Levels of IL-17 in the liver of untreated and DEM-treated *M.tb*-infected C57BL/6 mice; (**A**) 2 weeks post-infection, (**B**) 4 weeks post-infection, and (**C**) 8 weeks post-infection. The sample size (n) includes three female mice (n = 3) each in the untreated and DEM-treated groups. Dots represent replicates of each mouse. Comparisons between untreated and DEM-treated groups were analyzed via GraphPad prism software using an unpaired *t*-test with Welch’s correction. The placement of an asterisk (*) indicates statistical significance between compared groups. ns denotes non-significance. Double asterisks (**) denote a *p*-value < 0.005. Quadruple asterisks (****) denote a *p*-value < 0.0001.

Changes in the Th1 response following DEM treatment in *M.tb*-infected mice were monitored by assessing IL-12, IL-2, TNFα, and IFN-γ levels. A significant reduction in IL-12 in DEM-treated mice was observed 2, 4, and 8 weeks post-infection compared to untreated mice (Figure 4A–C). Compared to untreated mice, DEM treatment of *M.tb*-infected mice caused a significant 35% increase in the levels of IL-2 at 8 weeks post-infection and treatment. No changes were observed at the 2- and 4-week time points (Figure 4D–F). When compared to untreated mice, DEM treatment of *M.tb*-infected mice caused a significant increase in the levels of TNF-α at 2 weeks and 4 weeks post-infection and treatment (Figure 4G–I). No changes were observed at the 8-week time point. When compared to untreated mice, DEM treatment of *M.tb*-infected mice caused a significant increase in the levels of IFN-γ at 2 weeks and 8 weeks post-infection and treatment. The increase was almost two-fold. No significant changes were observed at the 4-week time point (Figure 4J–L). Th17 response during DEM treatment was assessed by measuring levels of IL-17. DEM treatment demonstrated no significant changes 2 weeks post-infection (Figure 5A). At 4 weeks post-*M.tb* infection, a significant decrease in IL-17 levels was detected in DEM-treated mice compared to untreated mice (Figure 5B). A significant decrease in IL-17 levels was also detected 8 weeks post-*M.tb* infection (Figure 5C).

**Figure 6:**
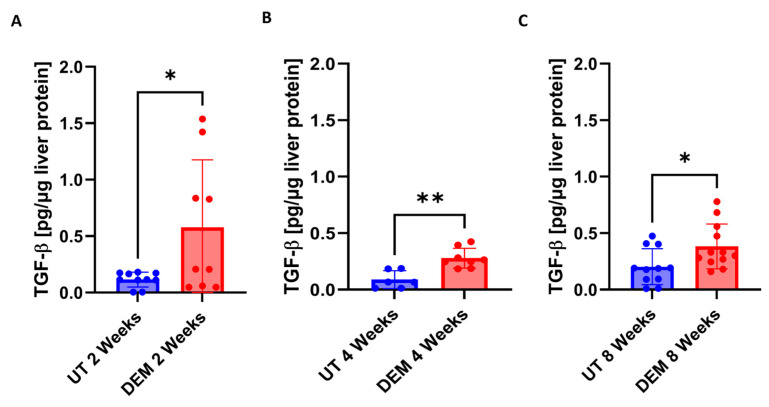
Levels of TGF-β in the liver of untreated and DEM-treated *M.tb*-infected C57Bl/6 mice; (**A**) 2 weeks post-infection, (**B**) 4 weeks post-infection, and (**C**) 8 weeks post-infection. The sample size (n) includes three female mice (n = 3) each in the untreated and DEM-treated groups. Comparisons between untreated and DEM-treated groups were analyzed via GraphPad prism software using an unpaired *t*-test with Welch’s correction. The placement of an asterisk (*) indicates statistical significance between compared groups. A single asterisk (*) denotes a *p*-value < 0.05. Double asterisks (**) denote a *p*-value < 0.005.

Immunosuppressive cytokine TGF-β was also assessed following the DEM treatment of *M.tb*-infected mice. DEM treatment of *M.tb*-infected mice led to a significant 6-fold increase in TGF-β levels at 2 weeks post-infection (Figure 6A). At 4 weeks post-*M.tb* infection, DEM treatment led to a significant 3-fold increase in TGF-β levels (Figure 6B). A significant 2-fold increase in TGF-β levels was also detected 8 weeks post-*M.tb* infection and DEM treatment (Figure 6C).

### 3.4. DEM Treatment Enhances M.tb Survival in the Liver and Spleen

**Figure 7:**
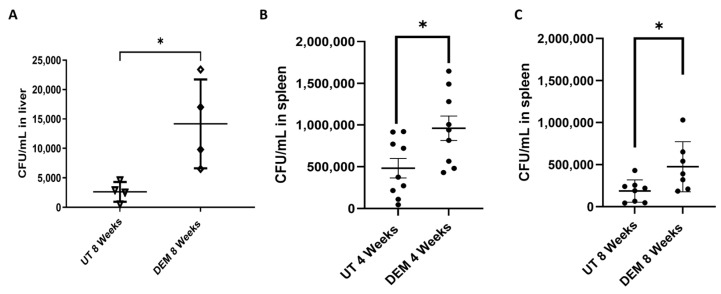
Survival of *M.tb* in the liver and spleen of untreated and DEM-treated *M.tb*-infected C57BL/6 mice. (**A**) CFU/mL *M.tb* in the liver of *M.tb*-infected mice 8 weeks post-infection. (**B**) CFU/mL *M.tb* in the spleen of *M.tb*-infected mice 4 weeks post-infection. (**C**) CFU/mL *M.tb* in the liver of *M.tb*-infected mice 8 weeks post-infection. The sample size (n) includes three female mice (n = 3) each in the untreated and DEM-treated groups. Dots represent replicates of each mouse. Comparisons between untreated and DEM-treated groups were analyzed via GraphPad prism software using an unpaired *t*-test with Welch’s correction. The placement of an asterisk (*) indicates statistical significance between compared groups. A single asterisk (*) denotes a *p*-value < 0.05.

DEM treatment led to a significant twofold increase in hepatic *M.tb* burden at 8 weeks post-*M.tb* infection (Figure 7A). Additionally, DEM treatment led to significantly increased *M.tb* burden in the spleen 4 and 8 weeks post-*M.tb* infection (Figure 7B–C).

### 3.5. Granuloma Formation in the Liver Is Altered Post-DEM Treatment

**Figure 8:**
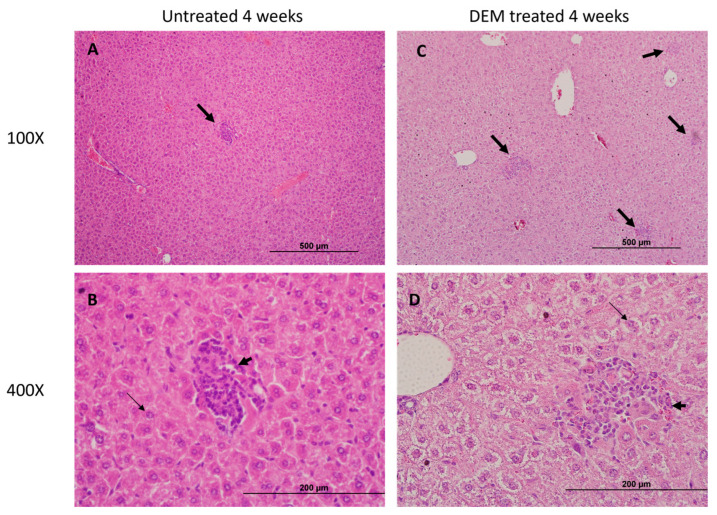
Hematoxylin and eosin (H&E) staining of the liver section of untreated and DEM-treated C57BL/6 mice 4 weeks post-*M.tb* infection. Thick arrows indicate immune cell infiltration into a granulomatous lesion; thin arrows indicate binuclear hepatocytes. Tissue sections were imaged at 100× and 400× the original magnification. (**A**) Hepatic tissue of untreated *M.tb* infected mice imaged at 100×. (**B**) Hepatic tissue of untreated *M.tb* infected mice imaged at 400×. (**C**). Hepatic tissue of DEM treated *M.tb* infected mice imaged at 100×. (**D**) Hepatic tissue of DEM treated *M.tb*-infected mice imaged at 400×. The scale bar for 100× panels refers to 500 µm, and for 400× panels, it refers to 200 µm.

**Figure 9:**
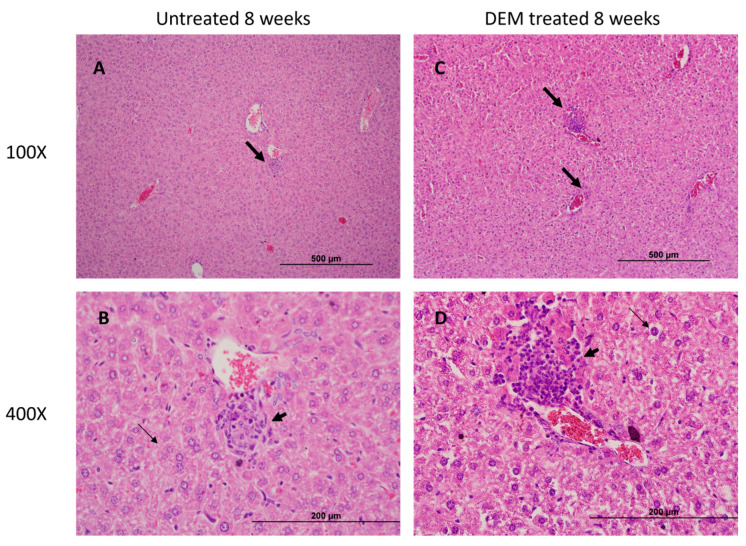
Hematoxylin and eosin (H&E) staining of the liver section of untreated and DEM-treated C57BL/6 mice 8 weeks post-*M.tb* infection. Thick arrows indicate immune cell infiltration into a granulomatous lesion; thin arrows indicate binuclear hepatocytes. Tissue sections were imaged at 100× and 400× the original magnification. (**A**) Hepatic tissue of untreated *M.tb* infected mice imaged at 100×. (**B**) Hepatic tissue of untreated *M.tb* -infected mice imaged at 400×. (**C**). Hepatic tissue of DEM treated M.tb infected mice imaged at 100×. (**D**) Hepatic tissue of DEM treated *M.tb*-infected mice imaged at 400×. The scale bar for 100× panels refers to 500 µm, and for 400× panels, it refers to 200 µm.

Well-defined intact and compact granulomas were observed in the liver of untreated mice at 4 and 8 weeks post-infection. In contrast to untreated, large-sized, diffuse granulomas were observed in DEM-treated mice at 4 and 8 weeks post-infection. (Figure 8A–D and Figure 9A–D).

## 4. Discussion

*Mycobacterium tuberculosis* (*M.tb*) primarily infects the lungs but can also affect other organs, leading to extrapulmonary TB. Immunocompromised patients, such as those with HIV or type 2 diabetes, are at increased risk of developing extrapulmonary TB [14,15]. Imbalances in redox homeostasis have been implicated in impaired host immune responses against TB [16]. Notably, patients with HIV and type 2 diabetes have diminished glutathione (GSH) levels and impaired granulomatous responses to *M.tb* infection [17,18]. The liver is one of the largest reservoirs for GSH production [19]. GSH serves essential functions in maintaining redox homeostasis, promoting antioxidant defenses, preventing lipid peroxidation, inhibiting ferroptosis, and detoxifying various metabolic processes in the liver [9]. Our laboratory previously elucidated the effects of GSH depletion during active TB in the lungs of *M.tb*-infected WT mice [12]. This study aims to examine the effects of hepatic GSH depletion during active extrapulmonary *M.tb* infection.

GSH exists in two forms: reduced glutathione (rGSH) and oxidized glutathione (GSSG) [20]. rGSH acts as an antioxidant by reducing reactive oxygen species (ROS) or reactive nitrogen species (RNS), forming GSSG in the process. GSSG is then converted back to rGSH via glutathione reductase and is coupled with NADPH to replenish functional GSH stores [20]. Diethyl maleate (DEM) is a maleate ester that depletes GSH by forming a covalent bond with the thiol group of rGSH, thus preventing its antioxidant function [21]. In this study, we utilized DEM to induce GSH-deficient conditions. DEM-treated mice showed reduced hepatic rGSH levels at 2 and 4 weeks post-*M.tb* infection, with undetectable levels at 8 weeks (Figure 1A). Conversely, GSSG levels remained significantly elevated throughout the study (Figure 1B).

Malondialdehyde (MDA) is a stable end product of cellular lipid peroxidation events and is a proxy for ROS injury [22]. We observed that DEM treatment compromised the antioxidant function of GSH in the liver, as indicated by elevated levels of MDA (Figure 2A–C). Furthermore, IL-6, an acute-phase cytokine produced in response to oxidative stress, was observed to be upregulated in DEM-treated mice (Figure 3A–C) [23]. GSH is also known to stimulate the Nrf2 pathway important in maintaining intracellular GSH levels [24,25]. DEM-induced GSH depletion can potentially affect the Nrf2-mediated intracellular GSH regulation and inhibit further GSH generation in macrophages. While we see a reduction in rGSH and an increase in GSSG levels following DEM treatment, the levels of GSSG do not significantly change over time, indicating stagnant generation of GSH and high oxidation of the existing pool.

*M.tb* infection is controlled by a Th1 cytokine response [26]. Kupffer cells, resident macrophages in the liver, recognize pathogen-associated molecular patterns from *M.tb* and phagocytose the pathogen as a first line of defense [27,28]. *M.tb* expresses a variety of virulent factors to prevent phagosome–lysosomal fusion and proliferates in infected cells [29]. The infected cells induce a Th1 cytokine response to recruit a diverse array of cells, including cytotoxic and helper T-cells, natural killer cells, monocytes, and neutrophils, to contain the infected cells within a granuloma [30]

Stimulated macrophages produce IL-12, promoting the differentiation of CD4+ T cells into Th1 cells [31]. Th1 cells then produce IFN-γ to enhance macrophage function and IL-2 to promote T-cell proliferation and further stimulate IFN-γ production [32]. Additionally, Th1 cells produce TNF-α to promote macrophage phagosome–lysosomal fusion, apoptosis, and recruitment of immune cells to the site of infection [33]. As the granuloma assembles, TH17 cells recruited at the site of infection produce IL-17 to enhance granuloma maturation [34]. TGF-β is an immunosuppressive cytokine secreted to regulate granuloma response [35].

GSH has been previously reported to stimulate Th1 cytokine production in macrophages, particularly IL-12 and IL-2 [24,36,37]. We hypothesized that diminished Th1 cytokine activation will result from rGSH diminishment. As expected, we observed a decrease in IL-12 with rGSH diminishment in DEM-treated mice (Figure 4A–C). However, surprisingly, IL-2 levels remained unchanged with DEM treatment with increased extrapulmonary *M.tb* infection and significantly increased at 8 weeks post-*M.tb* infection (Figure 4D–F). Additionally, IFN-γ levels were observed to be elevated following DEM treatment (Figure 4J–L). GSH depletion has previously been reported to reduce IFN-γ levels in APCs of mice [38]. These findings suggest IL-2 and IFN-γ are produced independent of Th1 cytokine induction by macrophages or GSH in the hepatic environment. Oxidative stress is known to activate transcription factors NFκB and AP-1 in which IL-6, TNF-α, IFN-γ, and IL-2 are expressed downstream, which could explain the observed elevated levels in this study [39]. More studies are needed to confirm these cytokine expression outcomes.

The granuloma is a hallmark of *M.tb* tissue pathology. In this study, DEM-treated mice exhibited larger granulomas with ill-defined borders compared to untreated mice (Figure 8A,D and Figure 9A,D). These findings are consistent with the elevation in TNF-α, suggesting increased recruitment of immune cells at the site of infection (Figure 4G–I). Additionally, a reduction in IL-17 levels in DEM-treated mice is consistent with the observed disorganized granuloma architecture in DEM-treated mice, indicating a lack of granuloma maturation (Figure 5B–C, Figure 8A–D, and Figure 9A–D). The granulomas were also associated with increased *M.tb* burden. *M.tb* infection was detected in the livers of both untreated and DEM-treated mice; however, GSH depletion resulted in a higher *M.tb* burden in the liver and spleen (Figure 7A–C). Together, the lack of a coordinated response due to rGSH depletion in these findings indicates a loss of granulomatous control of *M.tb*.

TGF-β is an immunosuppressive cytokine implicated in granuloma failure and tissue fibrosis [40]. TGF-β has been described to be a major inhibitor of cytotoxic T-cell effector function in granulomas, and deletion of TGF-β increases bactericidal activity by cytotoxic T-cells [41]. GSH has been described to quell the immunosuppressive effects of TGF-β by reducing ROS levels and stimulating SMAD7 production to inhibit TGF-β activation in SMAD2/3 signaling [42]. TGF-β levels were observed to be significantly elevated following DEM treatment, indicating loss of GSH-induced inhibition of TGF-β (Figure 6A–C). Along with increased hepatic and splenic *M.tb* burden and observed disorganized granuloma architecture, rGSH depletion promotes an immunosuppressive environment compromising host *M.tb* control. Despite elevated IFN-γ and IL-2 cytokine production observed in the study, reduced control of *M.tb* could be attributed to the TGF-β induced immune inhibition, reduced granuloma maturation, and increased tissue damage by oxidative stress.

Overall, DEM-induced rGSH depletion resulted in a dysregulated cytokine response, immunosuppression, and exacerbation of *M.tb* infection in extrapulmonary sites. This study provides further insight into the role of GSH in modulating the immune response against *M.tb* infections. This study is not without its limitations. Aspects of *M.tb* granuloma pathology could not be explored due to immune system variations in mice that differ from human TB pathology. Particularly, mouse granulomas tend to be less organized, lack a caseating core, and have with lesser extent of induced fibrosis [43]. Furthermore, the cellular origins of the cytokines assayed are unclear. Immunohistochemistry detailing the spatial relationships of the pro and anti-inflammatory cytokines on infected hepatic tissue, along with staining for *M.tb* bacilli distribution, can provide further correlations of areas of rGSH depletion, alterations in cytokine production, and *M.tb* control. Further investigation is needed to assess the Th1 response in the spleen. Further granuloma models should be explored to confirm the findings of this study. 

## 5. Conclusions

**Figure 10:**
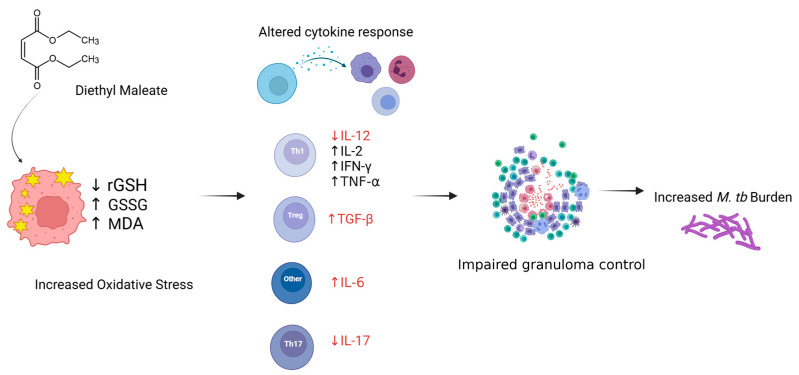
Overview of study findings: DEM-induced GSH depletion leads to an increase in MDA, dysregulated cytokine response, increased *M.tb* burden, and impaired granulomatous control of infection in the liver of WT C57BL/6 mice.

The findings of this study highlight the role of reduced GSH in modulating the immune response against extrapulmonary *M.tb* infection. The depletion of GSH through diethyl maleate (DEM) treatment led to significant oxidative stress, elevated pro-inflammatory cytokines, and impaired granulomatous responses (Figure 10). These changes resulted in increased *M.tb* burden and exacerbated extrapulmonary infection. The findings underscore the importance of maintaining redox homeostasis for effective immune defense against EPTB. Further research is needed to explore therapeutic strategies targeting GSH pathways to enhance host resistance to *M.tb*.

## Data Availability

The data supporting reported results can be obtained from the corresponding author (V.V.) upon formal requisition.
